# For post-graduation in urology: Is a preliminary degree in general surgery necessary?

**DOI:** 10.4103/0970-1591.70560

**Published:** 2010

**Authors:** Arabind Panda

**Affiliations:** Department of Urology, Christian Medical College Hospital, Vellore, India

**Keywords:** Urological training, integrated curriculum, medical education, residency programme

## Abstract

The format of urological training in India has changed little since its inception. The dogma of tradition has perhaps failed to consider the paradigm shifts in the science. A system that was relevant 50 years ago may not be so relevant today. The majority of procedures are endourological and laparoscopic, to which an average surgical resident has minimal exposure. Yet, the fundamentals of surgical craft are best learnt prior to any sub-specialty training. This is an apparent contradiction that has to be bridged if our training programs seek to be the foremost in the world. A single restructured training program that combines the core surgical curriculum to an extended exposure to the subspecialty will perhaps best address this issue.

## INTRODUCTION

Urological training in India in its present structure has changed little since its inception. Based on a modified Halstedian system, it requires a broad general surgical experience as a prerequisite for entry. Young urologists in training through a system of graded apprenticeship, involving increasing responsibility every year, safely acquire over 3 years, the necessary knowledge and skills. It is a system that has served us well, producing urologists of outstanding caliber.

The 3 years of general surgical training provides a comprehensive platform that allows a graduate to apply surgical principles to the treatment of disease. It produces a relatively undifferentiated, theoretically pleuripotent surgeon.[1] It is on such a frame around which further urological training is being imparted. The system has certain advantages. The exposure to trauma and emergency surgery is invaluable. Basic surgical principles, operating room decorum and dissection, resection and suturing techniques have already been ingrained in the urological trainee. Surgical intensive care training equips a surgeon with a working knowledge of intensive care, ventilators, and sepsis management. The handling of bowel and the principles of bowel reconstruction, so essential in reconstructive urology have been, in part already been taught. Most importantly, it is usually the exposure to urology as a junior resident that leads to increasing interest in the subject leading to its final choice as a career.[2]

## IS A FORMAL DEGREE IN GENERAL SURGERY NECESSARY?

Urology in India as elsewhere evolved from general surgery. Very competent general surgeons with a special interest in genitourinary surgery decided to specialize and were responsible for its growth in the initial years. Open surgery was mainly performed, and it was therefore of great benefit to have sufficient general surgical experience. Needless to say they also practiced general surgery. Times now have changed. An urologist now rarely practices general surgery. In fact many prefer to further sub-specialize within urology itself. Therefore, 3 years in general surgery may be too long for a person who has already decided to specialize in urology. A significant portion of urology is now endourology, which require an entirely different set of skills. Assisting and performing hepatobiliary resections does not beyond a point prove advantageous for a future urologist. Not all skills are transferred or transferable. An extended surgical training leading to a degree could be a waste of the most productive year of their lives, which could otherwise be dedicated to urology.[3] Furthermore, urology has dramatically evolved over the past two decades. Laparoscopic and endourological techniques have expanded both in scope and reach. As the molecular basis of urology becomes clearer, the need for a deeper understanding of the cellular and genetic basis of urological pathophysiology is apparent. The length of formal urological training arbitrarily fixed at 3 years, may not be sufficient for adequate urological competency. It becomes therefore necessary that urological trainees spend more time in specialty training to keep up with the expansion of relevant knowledge and technological advances. The perceived need is for a more comprehensive, focused education directed at the area of future practice.[1]

General surgery itself has expanded with increasingly complex surgical operations. The current training programs in surgery may be imparting a less complete degree of general surgery than in the past. The number of teaching cases in surgery is finite. It is believed that general surgery presently squanders precious teaching opportunities on residents who will practice in other fields while graduating incompletely trained general surgeons. These issues can be avoided with early specialization.[4] In this era of competition and inadequate opportunities for post graduation, it takes time and effort to be a general surgeon. To be an urologist is even more difficult, it being the most sought after subspecialty. Many years may elapse before a general surgeon qualifies as a urological trainee. There remains little doubt that a formal Master of Surgery (MS) degree before urological training wastes a few precious years of an urologists’ life for questionable benefit. It is perhaps time that urology evolves into a broad specialty from the subspecialty it is now, accepting students right after medical college, without a formal degree in surgery. This is a drastic change. Has it been done before? and most importantly will it work?

Urological training in various other countries follows an integrated urology residency model. In the United States residency programs in urology require 5 years of post-graduate education “which must include 36 months of clinical urology.” However, in practice almost all programs require 4 years of clinical and basic urology with a year of general surgery internship designed specifically for urology. The overall management of the training is by the American Board of Urology.[5] Australia and New Zealand have adopted in 2008 a new surgical education and training (SET) program involving early selection into specialty training. It is managed by the Urological Society of Australia and New Zealand (USANZ) under an arrangement with the Royal Australasian College of Surgeons (RACS). The management of the training program is the responsibility of the Board of Urology. SET Urology is a 6 year program involving basic and advanced surgical training in the first 2 years and then 4 years of clinical urology, the 6th year also being a fellowship year.[6] Canada has a similar model consisting of 2 years of core surgery training followed by a 3-year training period in urology. There is however no fellowship year.[7]

While appreciations of world trends are necessary, it is imperative that a urologic curriculum for India should take into account our unique demographical characteristics and needs.

## FACTORS THOSE ARE LIKELY TO IMPACT UROLOGICAL PRACTICE IN THE PRESENT AND FUTURE INCLUDE

### 

#### Increasing technological advances especially in minimally invasive urology

In the last decade, endourology has progressed rapidly. Lasers in many forms and robotics, apart from laparoscopy are being increasingly incorporated into urological practice. While some applications are extensions of previous techniques, most are recent innovations requiring a different set of skills and a steep learning curve.

#### Rapid development of information technology

Urology is now increasingly evidence based, and the internet is the cornerstone of evidence based urology. Information is now at our fingertips, that makes access to various medical database systems relatively cheap and within universal reach. Computer-based interactive case management systems in which a learner is presented with a virtual patient and asked to manage treatment have the ability to provide immediate feedback and identify areas of knowledge that need improvement.[1] The availability of faster dedicated broadband may see telesurgery progress beyond being just an experimental tool. Telementoring and telepresence have the potential to revolutionize urology.

#### Unwillingness to receive care from trainees and development of simulation technology

Apprenticeship as the core of postgraduate training, socially acceptable earlier may be less acceptable in the future. Over the last decade, simulation devices to teach hand and eye coordination have proliferated. The learning curve is more controlled without time limits, allowing objective evaluation of the ultimate result. It results in a measurable improvement in operating room efficiency, speed, and number of errors in comparison to traditional patient controlled models.[8]

#### The cost of equipment and training

New technology is expensive. Urology training programs need to have access to sufficient resources to impart currently relevant training, and to plan effective utilization of the expensive resources.

#### Increasing complexity and cost of basic and clinical urological research

Funding for medical research in India has always been meagre. With the increasing complexity of basic research and the extensive resources needed for clinical research, the teaching institutions may find it difficult to initiate and continue cutting edge research.

#### Encroachment of traditional specialty areas

Areas that were previously the primary domain of urologist, no longer remain so, e.g. the care of urological malignancies and female urology. With the increasing role of medical and surgical oncologists, change in treatment patterns, and increasing use of image-guided radiation and systemic therapy, the urologist may soon lose their status as the primary directors of care. More gynecologists are now training in advanced urogynaecology. It may have a significant impact on our care for women with benign conditions.

## PROPOSAL FOR AN ALTERNATIVE:THE NEW PARADIGM

### 

#### What should therefore a curriculum for urology in India include?

The Medical council of India in its “The Postgraduate Medical Education Regulations 2000” general conditions state that the curriculum shall be competency based, learning being essentially autonomous and self-directed. A modular approach is recommended for achieving a systematic exposure to various subspecialties. The training should involve learning experience ‘derived from’ and ‘targeted to’ the needs of the community. Furthermore, the goals should include mastery most of the competencies, pertaining to urology, that are required to be practiced at the secondary and tertiary levels of the health care delivery system.[9]

An ideal urology curriculum for India should:

- Serve specific needs of the patients and society.
- Be standardized throughout the country, establishing nationwide consistency in urological care.
- Incorporate basic science and clinical research.
- Maximize the educational component and minimize service without educational value.

#### A core of general surgery

While basic surgical principles are indispensible for any surgical sub-specialty training, a formal 3 year training program leading to a MS is unnecessary. It is however essentials that a shortened surgical program of 1–2 years specifically tailored for urology as the initial step. The curriculum must include a basic core of knowledge and experience needed to practice surgery, i.e. knowledge of wound healing, nutrition, infection, immunology, hemodynamics, and trauma.[1] It is imperative that it includes basic surgical techniques specially relating to the gastrointestinal tract.

#### Short tracking to an early specialization

Early exposure to the future specialty could provide a longer and better training.[2] The increasing complexity of operative urology and the expansion of the basic sciences and medical management mean that more time than the current 3 years are needed for competent urological training. A time frame of 4-5 years in clinical and basic urology seems more appropriate.

#### Collaboration and comprehensive training

All urological training programs in the country do not have sufficient volume in every aspect of urology to train all residents to competency in every sub-specialized field. Several training program may join together to provide trainees with an outstanding experience across the spectrum of urology.

#### Competency-based training

After the core surgical training, the rest of the urological residency could be competency based, i.e. allowing trainees to proceed through a program of escalating responsibility according to the competencies achieved. Exposure is not the same as experience and the student trainee must perform. The goal should be that competency is achieved when the trainee could perform the procedure independently or in the opinion of their teachers, they could be independent. This should apply not only to surgical techniques, but include all aspects of medical care including judgment.[10]

#### Incorporating clinical research

William Halsted and Harvey Cushing introduced the idea of the clinician scientist to modern medicine.[10] Research is an integral part of an urologist’s training. The goal should be to stimulate a lifelong interest in addressing questions that we all face on a daily basis. It shall also help to read the literature with a critical eye, with a solid understanding of study design, research methodology, and analysis.[11] Strong research programs result in increased prestige, improved recruiting of bright faculty, provide a scientific ground for residents and fellows apart from the opportunity for the creation of new knowledge.[12]

#### Newer techniques in surgical education

Urological training is currently is by apprenticeship. The techniques are intuitive and pedagogical. Perceptual learning, on the other hand, needs the student to take responsibility for his/her own education. The teacher serves to inspire, guide, and support the learning process. One of the goals of the training program should be to assure that self-directed learning methods are appreciated and employed, and the student pursues the knowledge to be gained and in the quest, often goes beyond what is formally included in the curriculum.[10] Training programs need to be more efficient, including more effective use of information technology, interactive systems, and simulation. Clear educational goals and learning objective need to be developed and appropriate end points established.[13]

#### The role of the mentor

The role model or the mentor is the single most important influence in choosing the specialty and for successful career development.[14] The choice of the specialty is often made at the junior resident level. Reorganization with shortening of general surgical training and greater emphasis on urology make it imperative that medical students are adequately counseled about subspecialties while in medical college, so they are in a position to make an informed choice; to meet mentors and role models who could guide them in the future. This may necessitate a slight modification of medical college curricula which now focuses on producing primary care physicians.

## THE REVISED STRUCTURE – A RECOMMENDATION FOR CHANGE

**Figure 1:**
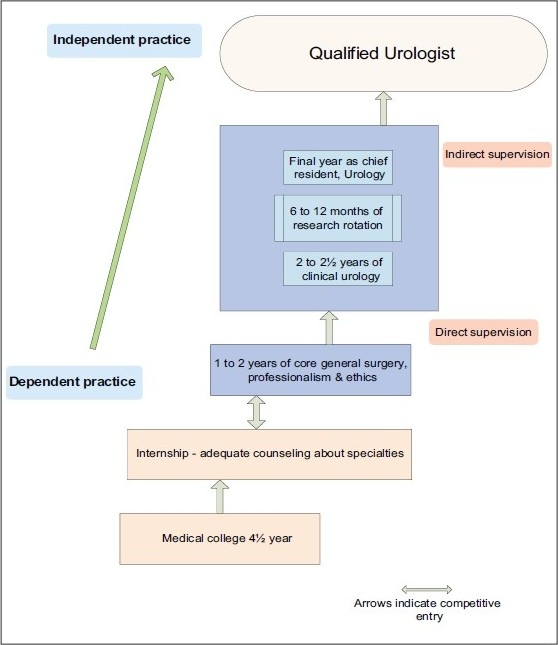
Proposed schema for restructured urology training in India

The recommendations outlined [Figure 1] here are deliberately ambitious, but achievable.

- The ultimate aim of all stages of medical education, including postgraduate training, is to improve the health and health care of the population. All policies need to be derived from this fact.
- Urological education in India needs to be regulated by a board of urology recommended by the Urological Society of India (USI). This board can be under the MCI and should be responsible for the structure, curriculum, and the overall quality of medical education.
- Entry can be after graduating from medical college, preferably through a common all India Entrance Examination which is fair and transparent.
- A formal MS degree is unnecessary. However, clinical urology training needs to be preceded by 1 or 2 years of core surgical training. The course has to be specifically tailored to the needs of urology. The content can be decided by the Board of Urology or the Urological Society of India in consultation with MCI. That the urology residents acquire the necessary skills during the surgical training will be the responsibility of the local urology department.
- Specific urology training can commence after the basic surgical training has been completed and should be for at least 4 years.
- Research is to be emphasized. Six months may be set aside in the 4 years as a research posting free from clinical duties. At the end of the training period, the trainee must publish his/her research or present it to an institutional research board to be eligible for the exit examination.
- Post-doctoral fellowship courses, as a part of a continuing professional developmental program, may be set up for those desirous of further training.

## CONCLUSIONS

Urological training in India has changed little since it began. Times change and knowledge increases. Training programs should evolve with both. The objective should be to redesign the training process to serve the need of patients, trainees, and society at large better.

Proposals for change almost always generate resistance. Modification to the present structure of training needs to be implemented carefully, methodically, and progressively to maximize benefits and minimize the trauma of transition. While the effects of early specialization on both urological and surgical workforce remains to be seen, there is little doubt that a formal degree is surgery is unnecessary. The success of a reorganized urologic curriculum is possible only if supported by the Urological Society of India and accepted by the Medical Council of India.

It is perhaps time to make the best better. The Halstedian concept of training has produced competent and safe urologists. However, the system as it stands today cannot cater to the need of the future urologist. We need to have a system that encourages competency, lifelong acquisition of knowledge, and its efficient, ethical and skillful application. Urological training is long and expensive for the society and in a variety of ways to the trainees themselves. The production of competent, safe practitioners should be our primary goal. We owe nothing less to our patients, profession, and society.
